# New York State Institute for the Study of Malignant Disease

**Published:** 1911-06

**Authors:** Harvey R. Gaylord

**Affiliations:** New York


### BUFFALO MEDICAL JOURNAL

**A Monthly Review of Medicine and Surgery**

*EDITOR*
WILLIAM WARREN POTTER, M. D.

All communications, whether of a literary or business nature, books for review and exchages, should be addressed to the editor, 238 Delaware Avenue, Buffalo, N.Y.

Vol. LXVI. JUNE, 1911. No. 11

### New York State Institute for the Study of Malignant Disease

THE enactment into law of the Loomis-LaReau bill for the establishment of the New York State Institute for the Study of Malignant Disease is the consummation of a fifteen years' campaign to commit the state to a definite policy of medical research. It is perfectly certain that with more advanced ideas regarding matters of public health the State of New York would ultimately have engaged in work of this kind. It has been, however, the vital nature of the cancer problem which has brought about this desirable end somewhat more rapidly than would have otherwise occurred. Through the initiative of Dr. Roswell Park and that splendid and enlightened support which this undertaking has enjoyed, notably from Mrs. Gratwick and from many other friends, both philanthropists and legislators, this institution is now definitely established by law in Buffalo.

The appropriation of $65,000 for a hospital will give to the Gratwick Laboratory the facilities which all modern institutes of medical research now either possess or are striving to acquire. Although this laboratory was the first to be established for the special study of cancer the institute in Heidelberg has for several years enjoyed both laboratory and hospital facilities. The Caroline Brewer Croft Cancer Fund at Harvard University has recently received a handsome gift from Mrs. Huntington for a small cancer research hospital with a sufficient endowment supplied by public-spirited citizens of Boston. The Rockefeller Institute which, in the scope of its work, covers all medical problems has a hospital in equipment and size in keeping with its large endowment and activities. Thus the time has come when the Gratwick Laboratory should have hospital facilities. At the same time it has been raised to the dignity of a state institution with a board of trustees and a defined policy of the state back of it.

The hospital will be for the accommodation of patients and will be conducted exclusively in the interests of science. Only such patients will be admitted as, through investigation, experimental treatment or otherwise, will prove of benefit to medical research. It will differ in many respects from established hospitals. The conduct of the entire institution, both laboratory and hospital, is placed by law in the hands of the director of the institute. The hospital will have no visiting staff. Cases will not be admitted except on order of the director for the purposes mentioned above. All cases will have it clearly explained to them on admission, that they are coöperating with the institution in the investigation of the particular disease with which they are afflicted; that they agree to all reasonable methods of treatment which the institute may propose.

The hospital will be equipped with every facility for investigation. Special arrangements will be made for the proper handling of sera and specimens of blood for examination. Various kinds of treatment will be tried out experimentally and, according to the act founding the institute, the results of investigation will be from time to time published.

It is the purpose of the director to give demonstrations for the benefit of the profession in the hospital. As the work of the institute enlarges opportunities will be afforded to qualified members of the profession to participate in the work of the institute. The established methods of treatment, including surgery, will be used in the hospital only as part of the general investigation of the disease. Although the hospital will be equipped with a suitable surgical operating room surgery as a means of treating cancer will not be given special consideration.

It is the belief of those interested in the institution that the foundation has now been laid for a state institute for medical research, which as time advances we believe may grow to larger dimensions and it is hoped that this center of research may prove a special stimulus to the profession of Buffalo and the State of New York. HARVEY R. GAYLORD.

